# Energy-Aware Control of Error Correction Rate for Solar-Powered Wireless Sensor Networks

**DOI:** 10.3390/s18082599

**Published:** 2018-08-08

**Authors:** Minjae Kang, Dong Kun Noh, Ikjune Yoon

**Affiliations:** 1 Department of Electronic Engineering, Soongsil University, Seoul 06978, Korea; mjkang@ssu.ac.kr; 2 Department of Software Convergence, Soongsil University, Seoul 06978, Korea; dnoh@ssu.ac.kr; 3 Department of Smart Systems Software, Soongsil University, Seoul 06978, Korea

**Keywords:** solar-powered, wireless sensor network, energy-aware, forward error correction, Reed–Solomon, blackout time, throughput

## Abstract

In a wireless sensor network (WSN) environment with frequent errors, forward error correction (FEC) is usually employed at the link layer to achieve reliable transmission. In the FEC scheme, the error correction rate varies depending on the length of parity used for the recovery of broken data. The longer the parity length, the higher the possible error correction rate. However, this also means that the energy consumption increases. Meanwhile, in a solar-powered WSN, the energy of each node can be periodically collected, but the amount of collected energy varies drastically depending on the harvesting environment, including factors such as the weather, season and time of day. Therefore, each node must control energy consumption according to the energy harvesting rate. The scheme proposed in this study executes this control by adaptively adjusting the parity length of FEC according to the given energy budget of a node for the next period. This means that the error recovery rate can be increased as much as possible without adversely affecting the blackout time. Simulation results show that the proposed scheme improves the amount of data collected from the entire network for each environment compared with previous schemes.

## 1. Introduction

Wireless sensor nodes consume limited hardware resources and have a short lifetime, especially owing to small battery capacities. For this reason, many studies are actively being conducted in order to overcome the problems of restricted energy in wireless sensor networks (WSNs). In particular, studies focusing on utilizing ambient energy to power WSNs are being carried out [1]. Among various ambient energies, solar energy is most frequently employed for WSNs because it not only has a high energy density, but also satisfies periodicity. However, because the amount of solar energy that can be harvested varies dynamically depending on the environment (e.g., time of day, weather, season and location), the energy consumption of a node must be carefully controlled with the consideration of the following objectives:

- Energy-neutral operation (ENO): The energy input during a harvesting period should not be less than the amount consumed during the same period. Because the energy harvested in one day can vary owing to environmental conditions, a node should adapt its power consumption rate to match the harvested energy for eternal operations.
- Minimizing the waste of harvested energy: In order to satisfy ENO, it is important to control the energy consumption such that it is below the energy input. This means that the less energy a node consumes, the better for satisfying the ENO conditions. However, in this case, a different problem can occur. Assuming that there is continuous sunny weather over several days, there may be a surplus of energy in excess of that required for a node’s general operation; thus, overcharged energy would inevitably be discarded owing to the capacity constraints of a rechargeable battery. Therefore, it is important to not only satisfy the ENO conditions, but also keep the remaining energy from exceeding the battery capacity.
- Reserving energy for the non-harvesting time: There is no energy to be harvested when the Sun has set. However, in many applications, the user wants to collect data at all times. Therefore, a node should reserve a certain amount of energy for the non-harvesting time. This requirement allows a node to operate regardless of the time of day.

Meanwhile, error control is typically performed for data transmission between nodes through an automatic repeat request (ARQ) or forward error correction (FEC) on a data-link layer. Using the ARQ method, data are re-transmitted if acknowledgment (ACK) is not delivered, as ACK indicates that data have successfully been received before timeout. On the contrary, in the FEC method, parity is generated by means of data encoding and then transmitted along with the data. If an error occurs when the target node receives these data, then the broken data can be restored by decoding the parity bits. In general, it is well known that errors are frequently generated in the channels of most WSNs [2,3]. Therefore, when the ARQ method is applied in a WSN environment, the number of retransmission requests increases, and thus, the amount of consumed energy increases proportionally. Therefore, the FEC method is more appropriate than the ARQ method for WSNs [4]. The FEC method restores errors based on the units of symbols, and the number of restorable symbols depends on the parity length. When a relatively long parity is applied, the number of restorable symbols becomes large. However, the amount of energy consumed also increases, and vice versa. Because of this, during FEC, the data-loss rate and energy-consumption rate have a trade-off relationship.

In this study, we attempt to improve the amount of data collected from a WSN by applying an adaptive FEC scheme to a solar-powered node. Note that we employ the Reed–Solomon (RS) method [5] among the many FEC schemes, since it has been shown to be one of the most efficient methods. On the basis of the RS method, we design an advanced energy-aware RS (EA-RS) scheme that adaptively adjusts the parity length of FEC according to the given energy budgets of both the source and target nodes for the next period. This means that the proposed scheme aims to increase the error recovery rate as much as possible while pursuing the three objectives for a solar-powered node described above. As a result, our efforts to enhance the data reliability never increase the blackout time of a node. In summary, EA-RS satisfies the following properties:

- Energy-adaptive operation: A node running EA-RS adjusts the energy consumption rate according to the energy collection rate by controlling the parity length used in the RS code. Thus, it can satisfy the ENO condition, minimize the waste of harvested energy and reserve energy for non-harvesting times, which leads to the best utilization of harvested energy.
- Enhancing the throughput of a WSN: Because the EA-RS utilizes surplus energy to increase data reliability (i.e., the error-correction rate), the amount of data collected at the sink during a time period can be increased.
- Fully-localized algorithm: A node running EA-RS only uses information for itself and its one-hop neighbors. This localized approach is crucially important for the scalability of a WSN.

The remainder of this paper is organized as follows. In Section 2, we describe the background of this research and review related work. In Section 3, we introduce our scheme for determining the parity length based on the solar-energy model. A performance verification of the proposed scheme through a simulation is presented in Section 4. Finally, we present our conclusions in Section 5.

## 2. Related Work

### 2.1. Solar Energy as a Power Source for WSNs

In the case of battery-based WSNs, most studies have attempted to overcome the limited battery problem by minimizing the energy consumption. However, even with this effort, the lifetime of a battery-based sensor node is usually under 30 days. Therefore, a periodic cost is required for network maintenance. Assuming that a WSN is used for applications that must be long-lasting, such as ecosystem management, forest fire detection or disaster monitoring, sensor nodes are supposed to be installed in dangerous or isolated locations that are difficult for a person to access. In these cases, the cost of maintaining the network would be significant. However, this problem can be resolved to some extent by using an energy harvesting sensor node, which collects energy from the environment, such as sun, wind or vibrations. Among these, solar energy is widely employed in WSNs, because of its higher harvesting rate (15 $\mathrm{mW}/{\mathrm{cm}}^{2}$) compared with other resources, as shown in Table 1. In addition, solar energy has the two following special properties:

- Periodicity: The Sun rises and sets once a day. This is the duration of a charging or harvesting cycle. A new supply of solar energy can be expected during every harvesting cycle.
- Dynamics: Solar energy varies throughout the day. Commonly, it increases in the morning, decreases in the afternoon and is absent during the night. In addition, it varies from day to day depending on the weather and season.

These characteristics necessitate a new energy optimization scheme that is suitable for solar-powered sensor nodes, which will be completely different from those appropriate for battery-based nodes. While a battery-based sensor node aims to minimize energy consumption, a solar-powered sensor node must attempt to fully utilize the periodically-harvested energy. In this study, we attempt to determine the best use of harvested energy, especially for data correction with the FEC scheme.

### 2.2. Energy Optimization for Solar-Powered WSNs

The first research issue that has been studied on energy optimization for solar-powered WSNs is to increase energy utilization in order to make the node operate constantly while utilizing most of the harvested energy. Kansal et al. [6] introduced an energy model for solar-powered nodes, which determines the bounds of energy that allow a node to survive indefinitely. Melodia et al. [7] proposed a transmission energy consumption model considering transmission distance, data length and path loss.

Then, the prediction of solar-energy harvested has been studied. The amount of energy harvested has to be accurately predicted and managed in order to ensure the maximized utilization. Kansal et al. [8] and Piorno et al. [9] introduced a harvested energy prediction scheme using a moving average approach and daily weather change, respectively. Cammarano et al. [10] proposed pro-energy, which can facilitate more precise prediction using long-term and short-term predictions according to weather and time.

Recently, the methods for allocating energy have been studied, in order to efficiently use time-variant solar energy independent of time. Noh et al. [11] proposed a scheme to allocate the necessary amount of energy to time slots considering the historic data according to time and weather. Zhang et al. [12] proposed a data gathering rate control scheme by allocating energy to the nodes and dynamically sensing and routing. Yoon et al. [13] proposed an adaptive data aggregation and compression to improve energy utilization. In this scheme, each node in a WSN periodically determines the amount of energy that it is allocated, which takes into account its residual energy and its likely acquisition and consumption. According to the allocated energy, it is determined whether the sensed data are aggregated with data from other nodes, compressed and transmitted. Our main goal is to allocate solar energy periodically to collect data stably and to use the surplus energy for recovering the broken data to improve data reliability.

### 2.3. ARQ and FEC in WSN Environments

The sensor nodes comprising WSNs have limited resources. Moreover, many nodes share a frequency bandwidth with other nodes. In terms of inter-node communication under the ZigBee protocol (IEEE 802.15.4), the resistance to noise is weaker than in heterogeneous wireless communication devices. For this reason, errors are highly likely to occur during inter-node communications in a WSN [2,3]. Moreover, as a WSN continually enlarges, inter-node interruptions and the probability of errors also increase, thereby reducing the entire throughput of the WSN. In this regard, the data-link layer, which ensures reliability, is crucial for inter-node communication in WSNs. Among many protocols for the data-link layer, the ARQ and FEC methods are primarily employed for inter-node communication.

In the case of successful data transmission, the ARQ method provides information through ACK that the data were successfully received, and it then requests subsequent data. However, when errors or data losses occur during data transmission, it requests data transmission again through a negative acknowledgment (NACK). Then, the data that failed in the original transmission are retransmitted instead of subsequent data. In contrast, the FEC method generates parity by encoding the data using an appropriate coding scheme (e.g., RS code) and transmits it along with the data. When errors occur, the broken data can be restored by decoding the parity using the same coding scheme.

**Figure 1:**
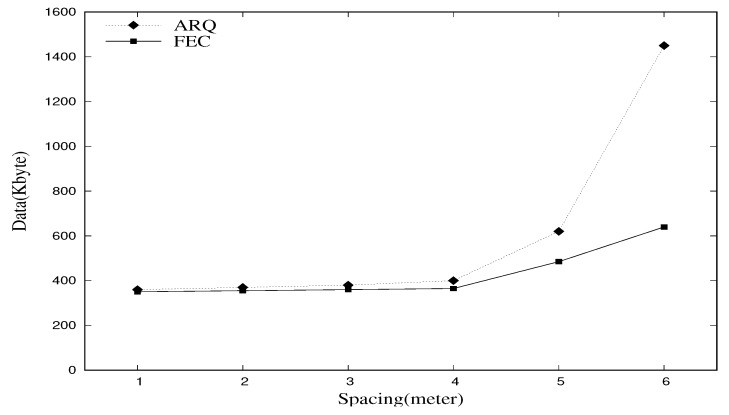
Total amount of data in the network required for the sink node to gather 80 KB of data. ARQ, automatic repeat request; FEC, forward error correction.

The graph in Figure 1 compares the total amount of data that should be transferred in a network in order to enable the sink node to gather all 80 KB of sensory data from each leaf node. This is performed through both the ARQ and FEC methods [14]. A balanced binary tree topology with eight leaf nodes was adopted in this experiment. Each leaf node contained 10 KB of sensory data. In Figure 1, the *x*-axis represents the spacing between nodes and the *y*-axis refers to the total amount of data generated (including retransmission data) in the entire WSN in order to reach 80 KB of sink-collection data. The spacing represents the physical distance between nodes, but it can also be thought of as an error rate, because the greater the distance, the higher the error rate. The results verify that the difference is insignificant when the spacing value ranges from one to four. A significant difference is evident when the spacing value is five or higher. Furthermore, as the spacing value increases, the values in the ARQ method increase in the form of an exponential graph, whereas those of the FEC method increase in the form of a linear graph. When the spacing value is six, the two methods exhibit a difference of more than twice that. Note that the increased amount of generated data has a negative influence on not only the energy consumption, as it uses much energy for data transmission, but also on the network performance, such as the throughput, because there is a higher chance of channels becoming occupied and congested. The results in Figure 1 confirm that the FEC method is significantly more efficient than the ARQ method for WSN environments in which errors are prone to occur. Consequently, most WSNs choose the FEC method as their link-layer protocol.

### 2.4. Reed–Solomon Block in the FEC Scheme

**Figure 2:**
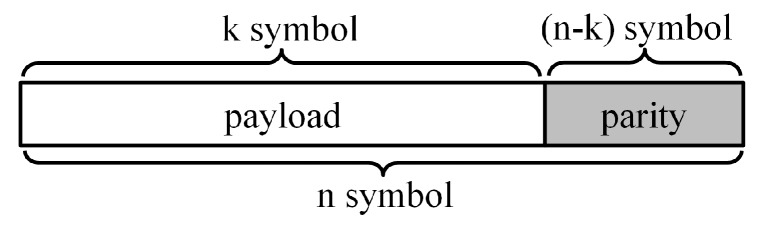
Reed–Solomon (RS) block.

Figure 2 shows an RS block used in an RS coding scheme, which is representative among the various FEC methods [5]. Data to be transmitted are included in the payload, and a code is inserted into the parity part, which the receiver will use in order to restore errors. If the RS block consists of *n* symbols in total, with *k* symbols in the payload and (n-k) symbols in the parity, then this is referred to as RS(n,k). It is well known that when an RS block of *n* symbols is transmitted, the maximum number of restorable symbols is (n-k)/2 [15,16,17].

Therefore, the number of restorable symbols can be adjusted by controlling the parity length in the RS block. If the parity length increases, then the number of restorable symbols also increases, and vice versa. On the contrary, in terms of energy consumption, an increase in the parity length inevitably leads to an increase in the energy required for encoding, decoding, transmitting and receiving data [15,16,17]. Table 2, which shows the amount of energy consumed for RS encoding and decoding for different parity lengths [15], supports this fact. This means that the energy efficiency and number of restorable symbols have a trade-off relationship.

## 3. Energy-Aware Reed–Solomon Scheme

As described in Section 1, a solar-powered node should satisfy the ENO requirements in an effort to not exhaust all energy (so as to avoid blackout). In addition, it should minimize the amount of energy that is discarded owing to the limited battery capacity and also reserve energy for use during nighttime when energy cannot be harvested. In this section, we explain the proposed EA-RS scheme to address these problems, which dynamically adjusts the sizes of RS blocks using the prediction of the energy input and output on solar-powered sensor nodes.

### 3.1. Overview of Node Operation with EA-RS

A node invokes EA-RS at the start of every data-transmission period, denoted as $p_{\mathrm{tx}}$, and repeats the following operations:

1. Energy prediction (explained in Section 3.2)
  - Prediction of harvested energy: Predict the energy harvested in the next period $p_{\mathrm{tx}}$ using the given solar-energy harvesting model [18]
  - Prediction of consumed energy: Predict the energy consumed in the next period $p_{\mathrm{tx}}$ with a varying parity length:
2. Determination of the parity length (explained in Section 3.3)
  - Determination of the parity length for the node itself: Calculate the appropriate parity length for $p_{\mathrm{tx}}$, considering its energy prediction
  - Finalization of the parity length: Finalize the appropriate parity length for $p_{\mathrm{tx}}$, considering the parity length of the target node
3. Data processing (explained in Section 3.4)
  - Sensing the data: Gather the data that the application requires
  - Encoding the data: Encode the sensory data to the RS block with the determined parity length
  - Transferring the data: Send the encoded RS block to the RF module at the physical layer for transferring

**Figure 3:**
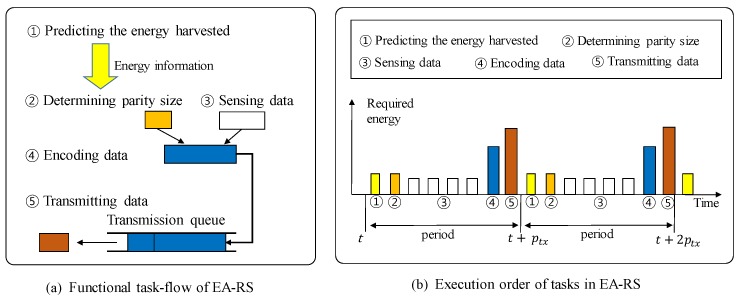
Overview of energy-aware (EA)-RS.

Figure 3 describes these operations in detail. Figure 3a describes the functional relationship between the operations described above, and Figure 3b shows the order in which the operations are performed in each period. By performing these operations, EA-RS can increase the data reliability of each node as much as possible within the available energy budget. Note that because the proposed method is based on predicting the energy consumptions of nodes, it can be applied more practically in time-based periodic applications, such as environmental monitoring applications, rather than event-based applications, which make it difficult to predict energy consumption.

### 3.2. Energy Prediction

In this section, we explain the energy models of $E_{\mathrm{harvest}}^{i}$ and $E_{\mathrm{consume}}^{i}$, which represent the predicted amount of harvested and consumed energy, respectively, for the node *i* during a given period.

Assume that the current period is the *n*-th period.

#### 3.2.1. Energy Harvesting Model

For a node *i* to obtain the amount of its energy expected to be harvested in the next period, denoted by $E_{\mathrm{harvest}}^{i}$, we employ the model of solar energy-harvesting prediction proposed in our previous work [18]. In this energy model, $E_{\mathrm{harvest}}^{i}$ can be calculated using historical information on the amount of energy actually harvested during each period. Specifically, each node measures and records $E_{\mathrm{harvest}}^{i}$, which is the actual energy harvested during the last period, and then $E_{\mathrm{harvest}}^{i}$ is updated using this information. $E_{\mathrm{harvest}}^{i}$ can be updated using a moving-average algorithm, as follows:

(1) $$E_{\mathrm{harvest}}^{i}\;⇐\;\left(1-\theta \right)\cdot E_{\mathrm{harvest}}^{i}+\theta \cdot E_{\mathrm{harvest}}^{i}$$

where *i* is the index of a node and θ is a real constant value between zero and one. θ is called the forgetting factor since it means the rate at which old measurements are forgotten. Note that a value of θ closer to one is used when the meaning of the present value is much more important than the past values, and a value closer to zero is selected when the present value is meaningful similarly to the past values. For example, if θ is 0.9, when calculating the moving average of the collected energy, the reflection ratio of the most recently-measured $E_{\mathrm{harvest}}^{i}$ is 0.9, the reflection ratio of the measured value in the preceding stage is 0.09, the reflection ratio of the measured value in the previous stage is 0.009, and so on. On the other hand, if θ is 0.1, the reflection rate starts at 0.1 for the most recent value of $E_{\mathrm{harvest}}^{i}$ and decreases to 0.09, 0.081, and so on. This means that the higher value of forgetting factor θ removes the influence of past values more quickly, and vice versa. When an energy collection system designer decides the value of θ, the amount of energy recently harvested is more meaningful than the past value since it reflects a short-term environment such as weather, but the past values also have some meaning because they reflect the long-term environment such as a season. Therefore, 0.5 is usually used as the value of θ for solar-powered WSNs.

#### 3.2.2. Energy Consuming Model

The energy consumption of a sensor node $E_{\mathrm{consume}}^{i}$ is generally modeled as follows:

(2) $$E_{\mathrm{consume}}^{i}=E_{\mathrm{transceiving}}^{i}+E_{\mathrm{system}}^{i}+E_{\mathrm{sensing}}^{i}$$

where $E_{\mathrm{transceiving}}^{i}$ and $E_{\mathrm{sensing}}^{i}$ are the amounts of energy consumed for data transceiving and data sensing, respectively, and $E_{\mathrm{system}}^{i}$ is the energy used in the remainder of the system other than data transceiving and sensing. At this time, $E_{\mathrm{transceiving}}^{i}$ can be expressed as the sum of the energy consumed for data transmission $E_{\mathrm{Tx}}^{i}$ and that used for data reception $E_{\mathrm{Rx}}^{i}$. In addition, $E_{\mathrm{system}}^{i}$ is divided into the FEC-related part and the non-related part as follows:

(3) $$E_{\mathrm{system}}^{i}=E_{\mathrm{FEC}\_\mathrm{sys}}^{i}+E_{\mathrm{general}\_\mathrm{sys}}^{i}$$

where $E_{\mathrm{FEC}\_\mathrm{sys}}^{i}$ refers to the FEC-related energy consumed by the system for encoding and decoding error recovery data, and $E_{\mathrm{general}\_\mathrm{sys}}^{i}$ is the energy used by the system excluding that related to FEC. Therefore, $E_{\mathrm{consume}}^{i}$ in Equation (2), which represents the total consumed energy of a sensor, can be summarized as follows:

(4) $$\begin{array}{c} E_{\mathrm{consume}}^{i}=E_{\mathrm{Tx}}^{i}+E_{\mathrm{Rx}}^{i}+E_{\mathrm{FEC}\_\mathrm{sys}}^{i}+E_{\mathrm{general}\_\mathrm{sys}}^{i}+E_{\mathrm{sensing}}^{i} \end{array}$$

The energy consumption terms we are interested in from Equation (4) are $E_{\mathrm{Tx}}^{i}$ and $E_{\mathrm{FEC}\_\mathrm{sys}}^{i}$, because these depend on the error recovery rate of FEC (which means the parity length of the RS block). The remaining energy consumption terms are unrelated to this and have almost fixed values. Therefore, from now on, we focus on modeling $E_{\mathrm{Tx}}^{i}$ and $E_{\mathrm{FEC}\_\mathrm{sys}}^{i}$ in detail.

The energy $E_{\mathrm{Tx}}^{i}$ consumed for data transmission depends on both the amount of data $D_{\mathrm{Tx}}^{i}$ to be transmitted and the transmission distance $d_{i}$. It can be expressed as follows [7]:

(5) $$E_{\mathrm{Tx}}^{i}=D_{\mathrm{Tx}}^{i}\cdot \beta \cdot {\left(d_{i}\right)}^{\alpha }$$

where β is the energy consumed to transfer one bit of data by one meter distance ($J/b/m^{\alpha }$) and α is the path loss, which is a natural number with a value of two or higher, depending on the transmission environment. Furthermore, $D_{\mathrm{Tx}}^{i}$ is the total amount of data that must be delivered by node *i*, including the amount of data it has sensed ($D_{\mathrm{sensing}}^{i}$), the amount of data that is received from neighboring nodes that node *i* must relay ($D_{\mathrm{relay}}^{i}$), and the parity length resulting from RS encoding ($D_{\mathrm{parity}}^{i}$). For the sake of convenience, let the sum of $D_{\mathrm{sensing}}^{i}$ and $D_{\mathrm{relay}}^{i}$ be called $D_{\mathrm{data}}^{i}$. Therefore, $D_{\mathrm{Tx}}^{i}$ can be expressed as follows:

(6) $$D_{\mathrm{Tx}}^{i}=\left(D_{\mathrm{sensing}}^{i}+D_{\mathrm{relay}}^{i}\right)+D_{\mathrm{parity}}^{i}=\left(D_{\mathrm{data}}^{i}+D_{\mathrm{parity}}^{i}\right)$$

By using Equation (6), Equation (5) can be summarized as follows:

(7) $$E_{\mathrm{Tx}}^{i}=\left(D_{\mathrm{data}}^{i}+D_{\mathrm{parity}}^{i}\right)\cdot \beta \cdot {\left(d_{i}\right)}^{\alpha }$$

Moreover, $E_{\mathrm{FEC}\_\mathrm{sys}}^{i}$ includes the RS encoding and decoding energy and is expressed as follows:

(8) $$\begin{array}{c} E_{\mathrm{FEC}\_\mathrm{sys}}^{i}=E_{\mathrm{encoding}}^{i}\left(D_{\mathrm{data}}^{i},D_{\mathrm{parity}}^{i}\right)+E_{\mathrm{decoding}}^{i}\left(D_{\mathrm{relay}}^{i},D_{\mathrm{parity}}^{i}\right) \end{array}$$

$E_{\mathrm{encoding}}^{i}\left(A,B\right)$ and $E_{\mathrm{decoding}}^{i}\left(A,B\right)$ represent the amounts of energy consumed for operation and storage when encoding and decoding, respectively, data of size *A* using the parity length *B*. It should be noted that the total amount of data that the sending node *i* must encode is $D_{\mathrm{data}}^{i}$, which contains not only the data it has sensed ($D_{\mathrm{sensing}}^{i}$) but also the data received from other nodes ($D_{\mathrm{relay}}^{i}$).

Again, it is important to note that of the total energy $E_{\mathrm{consume}}^{i}$ (Equation (4)) used by node *i*, the terms related to the error recovery rate of FEC (i.e., the parity length of the RS block) are $E_{\mathrm{Tx}}^{i}$ (Equation (7)) for data transmission and $E_{\mathrm{FEC}\_\mathrm{sys}}^{i}$ (Equation (8)) for data encoding and decoding with the RS scheme.

### 3.3. Determining the Parity Length for the Next Period

In this section, we describe how the EA-RA scheme dynamically determines the parity length for data reliability according to the amount of collected energy. For example, suppose that a node always transmits data using the maximum parity length. The node is likely to consume more energy than the amount of energy harvested during a period. In this case, the node violates ENO conditions, and thus, it is likely to suffer from blackout, especially during the nighttime. However, the problem of overcharging (inevitably causing dissipation) energy owing to the limited battery capacity will not occur. Moreover, a node that transmits data with a minimum rate of recovery (minimum parity length) can save much energy in the rechargeable battery (reducing the blackout time), but it is possible that there will be energy that is still dissipated owing to the limited battery capacity. To address this trade-off problem, the EA-RA scheme dynamically adjusts the data recovery rate in order to minimize the blackout time while also minimizing the energy dissipation owing to battery capacity limitations.

#### 3.3.1. Determining the Parity Length for a Node Itself

In EA-RS, determining the parity length consists of predicting the amounts of energy collected and consumed. In short, it chooses a parity length that satisfies the following two requirements:

- (1) The parity length that allows the amount of residual energy after the end of the next period not to exceed the maximum capacity of the battery: these parity lengths can minimize the amount of energy dissipated owing to the limited battery capacity. By using these parity lengths, the node consumes more energy than must be wasted in order to prevent the remaining energy from exceeding the battery capacity.
- (2) The smallest parity length among those of (1): by using this parity length, the node can use the minimal required energy for data reliability, except for energy that must be wasted. That is, it allows the node to store as much energy as possible while minimizing the blackout time.

As described above, this parity length is the optimal value in order to minimize the blackout time while also minimizing the energy that is discarded owing to the constrained battery capacity, and EA-RS should determine this value.

To determine the parity length, the predicted amount of residual energy after the next period should be modeled. If the current time is *t* and the transmission period is denoted by $p_{\mathrm{tx}}$, then the estimated amount of energy remaining in node *i* after this period, $E_{\mathrm{residual}}^{i}\left(t+p_{\mathrm{tx}}\right)$, can be modeled as follows [18]:

(9) $$E_{\mathrm{residual}}^{i}\left(t+p_{\mathrm{tx}}\right)=E_{\mathrm{residual}}^{i}\left(t\right)-E_{\mathrm{consume}}^{i}+E_{\mathrm{harvest}}^{i}$$

where $E_{\mathrm{residual}}^{i}\left(t\right)$ denotes the amount of energy remaining in the node at the current time *t*, which can be obtained through an actual measurement, and $E_{\mathrm{consume}}^{i}$ and $E_{\mathrm{harvest}}^{i}$ are the predicted amounts of consumed and collected energy, respectively, during the period $p_{\mathrm{tx}}$ from time *t* to time $t+p_{\mathrm{tx}}$, which can be obtained through Equations (1) and (4). In particular, as can be seen from Equations (7) and (8), the energy consumption $E_{\mathrm{consume}}^{i}$ can be controlled by varying the parity length. Our goal is to find the optimal parity length to store the maximum amount of energy in order to reduce blackout time while minimizing the energy dissipation owing to battery capacity limitations.

To achieve this, we gradually increase the parity length $D_{\mathrm{parity}}^{i}$ starting from the minimum value and repeatedly verify that it satisfies the following Equation (10). That is, the initial parity length value (minimum parity length) should first be tested so that the expected remaining energy $E_{\mathrm{residual}}^{i}\left(t+p_{\mathrm{tx}}\right)$ of node *i* is calculated according to Equation (9). Then, this is compared with the maximum capacity $B_{\mathrm{full}}^{i}$ of the node *i*. If the following Equation (10) is satisfied, then the parity length $D_{\mathrm{parity}}^{i}$ is increased by γ.

(10) $$\begin{array}{c} E_{\mathrm{residual}}^{i}\left(t+p_{\mathrm{tx}}\right)>B_{\mathrm{full}}^{i} \end{array}$$

where γ is the factor by which the parity length is increased, which is a natural number. Increasing $D_{\mathrm{parity}}^{i}$ increases the data recovery rate, but also increases the node’s energy consumption, to reduce the amount of remaining energy. This procedure is repeated until Equation (10) cannot be satisfied. As a result, EA-RS can obtain $D_{\mathrm{parity}}^{i}$, such that the maximum energy can be stored while minimizing the wasted energy. The minimum parity length will be selected for periods with no energy collection (e.g., nighttime). ENO can never be satisfied for such periods, regardless of how short the parity length is, as the amount of collected energy is less than the energy required for the node to perform only basic operations (excluding FEC operations). Therefore, even the minimum parity length cannot satisfy Equation (10), so the minimum parity bit value is selected as it is. In this case, the node attempts to conserve the energy consumption by minimizing the work for data recovery.

#### 3.3.2. Finalizing the Parity Length Considering a Target Node’s Status

When the node sends data, the parity length of the receiving node must be considered. Note that the amount of energy used by the receiving node to decode the data depends on the parity length, as shown in Table 2, and the difference is not negligible. Assume that the sending node transfers the data encoded with its own parity length, which is larger than that of the receiving node. Then, the receiving node would consume more energy than it anticipates when performing the decoding and could be blacked out, because the receiving node predicts the amount of energy consumed in decoding with assuming that the incoming data would be encoded with its own parity length instead of the sender’s one. In this case, therefore, the parity length of the sending node and the receiving node should be compared, and if that of the receiving node is smaller, the parity length of the sending node must be reduced to that of the receiving node.

For this reason, in order to determine the final parity length, we must consider not only the node’s own parity length $D_{\mathrm{parity}}^{i}$ but also the parity length $D_{\mathrm{parity}}^{j}$ of the receiving node *j*. To this end, the transmitting node must know the parity length of the receiving node $D_{\mathrm{parity}}^{j}$, which is possible by receiving this from the neighbor node *j* at the start of every period. Such an operation can be implemented by including the information on the parity length in the routing control packet. This increases the size of the routing control packet. However, because the parity length is a small natural number, the overhead is negligible. At node *i*, EA-RS compares the parity lengths of itself and the receiving node *j* in order to determine the final parity length of node *i* as follows:

(11) $$D_{\mathrm{parity}}^{i}<D_{\mathrm{parity}}^{j}$$

If Equation (11) is satisfied, then the data are encoded and transferred with the parity length of $D_{\mathrm{parity}}^{i}$, because the receiving node *j* has sufficient energy to handle it, which means that the blackout time of node *j* will not increase. In the opposite case (if Equation (11) is not satisfied), the node *i* encodes and transmits the data with the parity length $D_{\mathrm{parity}}^{j}$, as the blackout time of the receiving node *j* increases when the transmitting node *i* encodes and transfers data with its current parity bit size $D_{\mathrm{parity}}^{i}$.

In summary, because the energy consumption of both the transmitting and receiving nodes becomes larger as the parity length increases, the transmitting node selects a lower value between the parity lengths of the transmitting and receiving nodes. Then, the data are encoded and transferred with this lower parity length so that the blackout times of both nodes are unaffected. In this manner, EA-RS dynamically adjusts the parity length to the best energy consumption level for improving data reliability. That is, EA-RS uses as much energy as possible in order to increase the data recovery rate, but the energy used for this operation does not cause additional blackout time, because it only uses energy that would be discarded owing to the limited battery capacity. The pseudo-code of the proposed EA-RS algorithm is described in Algorithm 1.

**Algorithm 1:** EA-RS(*i*)
**1**
$D_{\mathrm{parity}}^{i}$⟵ 1;
**2**
$D_{\mathrm{parity}}^{j}$⟵ size of parity bits of node *i*’s target node;
**3**

**while**
$E_{\mathrm{residual}}^{i}\left(t+p_{\mathrm{tx}}\right)>B_{\mathrm{full}}^{i}$
**do**

**4**
 | $D_{\mathrm{parity}}^{i}⟵D_{\mathrm{parity}}^{i}+\gamma$;
**5**

**end**

**6**
Broadcast the $D_{\mathrm{parity}}^{i}$ to neighbor nodes using ACK packet;
**7**

**if**
$D_{\mathrm{parity}}^{i}<D_{\mathrm{parity}}^{j}$
**then**

**8**
 | Encoding with parity $D_{\mathrm{parity}}^{i}$;
**9**

**else**

**10**
 | Encoding with parity $D_{\mathrm{parity}}^{j}$;
**11**

**end**

**12**
sleep(period);

### 3.4. Architecture of EA-RS in a Sensor Node

**Figure 4:**
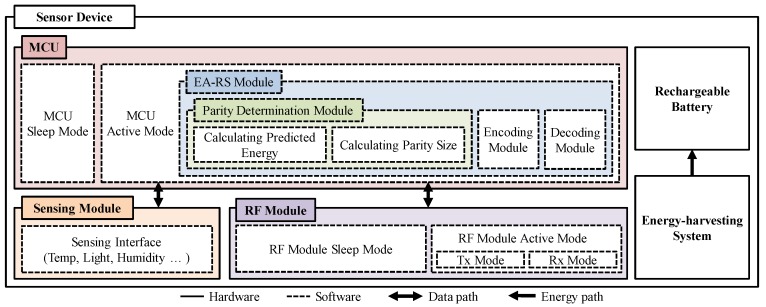
Architecture of a node in which the EA-RS method is applied.

Figure 4 depicts the sensor node architecture with the application of the proposed EA-RS method. The micro-controller unit (MCU) modes of sensor nodes are largely classified into sleep and active states. When an MCU is in sleep mode, the sensor node operation is limited in order to minimize the energy consumption. When the MCU is in active mode, practical tasks requiring sensor nodes are performed.

Required tasks are performed through various modules, such as sensing the various data required by the application through the sensing module and transceiving the data through the radio frequency (RF) module. The EA-RS module additionally operates when an MCU is in an active mode. This first invokes the parity-determination submodule in order to calculate the predicted residual energy and determine the parity length for the next period. This submodule is performed based on the historic information on energy collection and the predicted amount of energy consumption in the next period, as well as the parity lengths of neighboring nodes. Subsequently, the EA-RS module invokes the encoding submodule in order to create an RS code with the parity length determined at the parity-determination submodule, and then, this RS code is moved to the RF module in order to be transferred.

## 4. Experimental Results and Discussion

We compared RS(36, 32) and RS(68, 32) and the recently-proposed two-level RS scheme [19] with EA-RS for a performance verification. RS(36, 32) uses only four symbols (32 bits) as parity bits for the error recovery of 32 data symbols (256 bits) in an RS block. This RS(36, 32) enjoys the advantage of the amount of energy used in the FEC being small, but the drawback is that the data recovery rate is also low. On the contrary, RS(68, 32) uses 34 symbols (272 bits) as parity bits for the error recovery of 32 data symbols (256 bits). Owing to the long parity bits, RS(68, 32) has the advantage of a high data recovery rate, but the amount of energy used is inevitably high. The two-level RS scheme is similar to EA-RS, in that the parity length is dynamically determined according to the energy state. However, unlike EA-RS, which determines parity lengths in further detail from minimum to maximum depending on the amount of energy, two-level RS selects one from only two schemes: RS(36, 32) and RS(68, 32).

### 4.1. Experimental Environment

**Figure 5:**
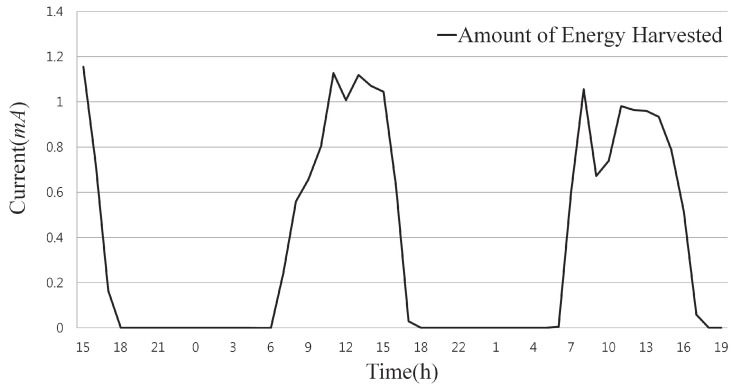
Sample part of energy input according to time.

The performance evaluation was carried out using Solar-Castalia [20], which is a simulator specially designed for solar-powered WSNs. The harvested solar energy used in the simulation was based on real data [21] taken from actual solar-powered motes. The weather varied in a cycle with a length of one day, and sunny, cloudy or rainy days were randomly selected. Figure 5 shows sample data of energy harvested over two days. For the moving average shown in Equation (1), the value of θ was set to 0.5 due to the characteristics of solar energy as explained in Section 3.2.1, and the update period was 1 h. The payload of the RS block was composed of 32 symbols, and the size of each symbol was 8 bits. That is, the payload size of the RS block was 256 bits. The sensing rate of each node was 256 bits per round. In addition, the nodes were randomly arranged and experimented with on various network sizes and error environments by varying the field size, number of nodes and error rate. Details of the experimental conditions can be found in Table 3.

### 4.2. Blackout Time Analysis

**Figure 6:**
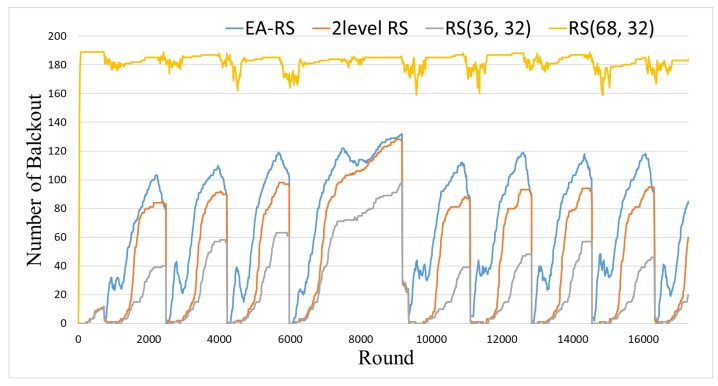
Number of blackout nodes for 200 nodes and 0.15 BER.

**Figure 7:**
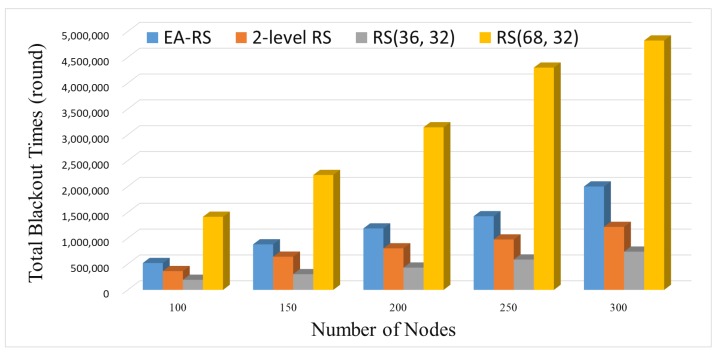
Blackout for varying number of nodes during the entire time (BER 0.15).

Figure 6 and Figure 7 present the results related to the blackout time. First, Figure 6 shows the number of blackout nodes over time for a total of 200 nodes with 0.15 BER (bit error rate). As can be seen in this figure, RS(68, 32) exhibits the largest number of blackout nodes during the entire time. This is because RS(68, 32) uses much energy to achieve the high error correction rate. On the contrary, RS(36, 32) has the lowest number of blackout nodes, which means that this scheme spends the lowest amount of energy on error correction. The two-level RS scheme and EA-RS yield similar results, which are considerably lower than RS(68, 32). This is because both schemes determine the error-correction rate dynamically by considering the energy state of a node.

The blackout time for each network was also measured while increasing the number of nodes. Figure 7 shows the results for the accumulation of all blackout time for each network, where the numbers of nodes are deployed. Note that because the results show the accumulated values of all nodes, it is natural that the blackout time increases as the number of nodes increases. The part we are most interested in is the linear form of increasing lines, especially for the EA-RS scheme. This means that even though EA-RS uses the most complicated algorithm among all schemes, the overhead per node does not change, regardless of the network size, because it is a fully-localized scheme. Assuming that a certain scheme is not localized, the sum of the blackout times will increase exponentially as the number of nodes increases, which means that the scheme has no scalability. Therefore, from the results of this experiment, it can be confirmed that the EA-RS scheme has a high scalability.

### 4.3. Received Data Analysis

**Figure 8:**
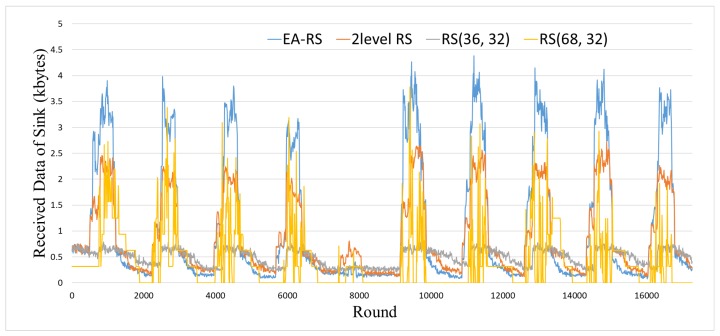
Received data according to time, for 200 nodes and 0.15 BER.

Figure 8 shows the total amount of data received by the sink node over time. This experiment was conducted in a BER 0.15 environment with 200 nodes. It can be seen that the network with RS(68, 32) achieves a higher instantaneous data collection rate than RS(36, 32) owing to its larger parity length, but it is impossible to collect data stably because the node is out of power for long periods. On the contrary, the data collection rate of the network with RS(36, 32) is more consistent than that of RS(68, 32), owing to the lower blackout time. However, the total amount of data collection is the lowest, because the error correction rate is too low to prevent the retransmission of broken data. Compared with RS(68, 32) and RS(36, 32), two-level RS and EA-RS collect data with higher rates most of the time. In particular, EA-RS achieves a higher amount of data collection than two-level RS, because it controls the parity length more finely and precisely.

**Figure 9:**
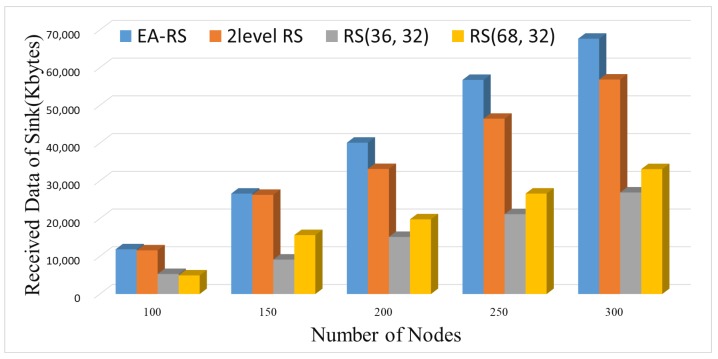
Received data of sink for varying number of nodes (BER 0.15).

In addition, we experimented with a different number of nodes in the same environment. Figure 9 shows the data collection results for the sink node according to the number of nodes. The amount of collected data for the EA-RS method is the highest in every network size, and the difference increases as the number of nodes increases.

### 4.4. Performance in Various Error Environments

**Figure 10:**
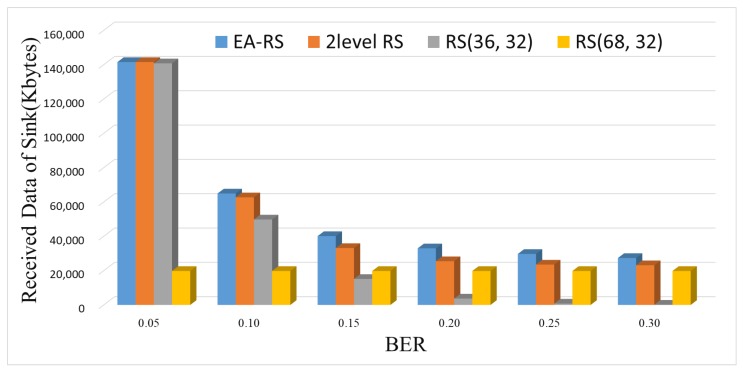
Received data of sink for varying BER (Node 200).

**Figure 11:**
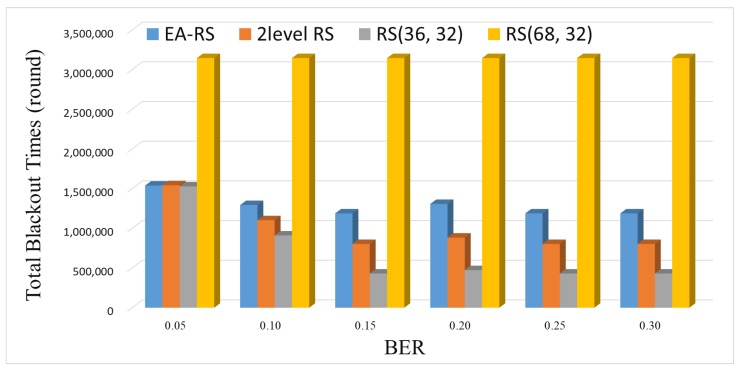
Blackout during entire time for varying BER (Node 200).

Figure 10 and Figure 11 show the amounts of data collected by a sink node and the blackout times, respectively, according to a varying BER. As can be seen from Figure 10, RS(68, 32) yields a constant amount of data irrespective of the BER, because data recovery is possible even if the channel condition is poor, owing to its high error correction rate. However, as can be seen from Figure 11, RS(68, 32) exhibits the greatest amount of blackout time at all BERs, which degrades the data collection of the sink.

Meanwhile, as shown in Figure 10, the three schemes of EA-RS, two-level RS and RS(36, 32) exhibit similarly high collection results when the BER is 0.05. This is because there is less need to recover data owing to the low BER. However, as the BER increases, the amount of collected data decreases. In particular, in the case of RS(36, 32), the amount of data collected at the sink is almost zero when the BER is 0.25 or greater.

EA-RS exhibits the strongest performance, even when the channel state deteriorates, because it finely and adaptively controls the error correction rate. When comparing EA-RS to two-level RS, we can see that EA-RS achieves a slightly higher (inferior) blackout time, as shown in Figure 11. However, this result does not mean that EA-RS does not perform better than two-level RS, but rather, it means that EA-RS conducts more aggressive control of the data recovery rate, which can lead to a large amount of data transmission. This can be confirmed by the fact that the data collection rate of EA-RS is the highest in all error environments in Figure 10. In other words, the EA-RS scheme can further improve the overall amount of data collection by adjusting the parity bits more finely than the previous two-level RS scheme.

## 5. Conclusions

As WSNs based on solar power can harvest energy periodically, they aim to optimize the energy consumption rather than minimize it. In this study, we control the reliability of the data transmission using the FEC scheme in order to optimize the energy consumption given that there are frequent errors in general WSN environments. In the FEC scheme, when a relatively longer parity is employed, broken data are more likely to be fixed, which leads to a decrease in the data loss rate, thereby increasing the reliability. However, the energy consumption can be larger, leading to increased blackout times. When using a shorter parity, the opposite results occur.

In this paper, we proposed EA-RS, which is a localized method for increasing the reliability of WSNs by using surplus energy without increasing blackout times. More specifically, EA-RS computes the amount of surplus energy that should be discarded owing to battery capacity limitations by predicting the energy collection and consumption of the next cycle, using only information about itself and its neighbors. Then, it adjusts the data recovery rate by fine-tuning the optimal parity length best suited to the energy status of itself and the target node. Through various experiments, we verified the effectiveness of the proposed method, which can enhance the reliability without adversely affecting the blackout time.

## Figures and Tables

**Table 1:** Types and characteristics of harvesting sources  (Data from [1]).

Energy Source	Characteristics	Amount of Energy Available	Harvesting Technology	Conversing Efficiency	Amount of Energy Harvested
Solar	Ambient, Uncontrollable, Predictable	100 mW/cm^2^	Solar Cells	15%	15 mW/cm^2^
Wind	Ambient, Uncontrollable, Predictable	-	Anemometer	-	1200 mWh/day
Finger motion	Active Human power, Fully controllable	19 mW	Piezoelectric	11%	2.1 mW
Vibrations in indoor environments	Ambient, Uncontrollable, Predictable	-	Electromagnetic Induction	-	0.2 mW/cm^2^

**Table 2:** Energy consumption of RS encoding and decoding (Data from [15]).

RS Code	Encoding Energy (mJ)	Decoding Energy (mJ)
(15, 13)	0.1844	0.2352
(31, 27)	0.3499	0.4281
(63, 55)	0.5631	0.6872
(127, 115)	0.8798	1.0354

**Table 3:** Experimental environment.

Parameter	Description
Time unit	round (1 round = 1 min)
Experimental time	43,200 round (30 days)
RF module	CC2420
Number of nodes	100, 150, 200, 250, 300
Size of field	3600∼6100 m${}^{2}$
Transmission range	10 m
Channel status (BER)	0.05, 0.10, 0.15, 0.20, 0.25, 0.30
Sensing rate	256 bits/round/node
Weather	Random
θ in moving average	0.5
Update period of moving average	1 h
Avg. harvesting energy per day	33.5 J
Energy consumption rate for transferring data	52.2 mJ/s
Energy consumption rate for receiving data	59.1 mJ/s
RS(36, 32) encoding energy	0.860 mJ/s
RS(36, 32) decoding energy	1.477 mJ/s
RS(68, 32) encoding energy	6.612 mJ/s
RS(68, 32) decoding energy	22.515 mJ/s
